# Discriminating Aroma Compounds in Five Cocoa Bean Genotypes from Two Brazilian States: White Kerosene-like Catongo, Red Whisky-like FL89 (Bahia), Forasteros IMC67, PA121 and P7 (Pará)

**DOI:** 10.3390/molecules28041548

**Published:** 2023-02-06

**Authors:** Sonia Collin, Toine Fisette, Anne Pinto, Jesus Souza, Hervé Rogez

**Affiliations:** 1 Unité de Brasserie et des Industries Alimentaires, Louvain Institute of Biomolecular Science and Technology (LIBST), Faculté des Bioingénieurs, Université catholique de Louvain, 1348 Louvain-la-Neuve, Belgium; 2 Centre for Valorization of Amazonian Bioactive Compounds (CVACBA), Universidade Federal do Pará (UFPA), Belém 66075-750, PA, Brazil

**Keywords:** cocoa, Brazil, flavors, whisky, Catongo, GC-EIMS, SAFE

## Abstract

Fine-grade cocoa beans are characterized by their great organoleptic quality. Brazilian cocoa producers increasingly privilege organoleptic quality over yield. Catongo and FL89, fine Brazilian cocoa genotypes from Bahia, are characterized by particular flavors (respectively, kerosene and whisky). The beans IMC67, PA121, and P7 from the state of Pará are genotypes that have high resistance to diseases. Solvent-assisted flavor evaporation (SAFE) aroma extraction was used here to identify and quantify the volatile compounds discriminating these genotypes. The results show that the kerosene aroma of Catongo is likely due to the presence, at high levels, of ethyl acetate and isobutyl acetate. On the other hand, ethyl benzoate, heptanoate, and octanoate, *trans*-2-nonenal, 1-octen-3-ol, and 3-methylbutanol could play a key role in the whisky notes of FL89. Heptan-2-ol, heptan-2-one, nonan-2-one, linalool (although still more concentrated in IMC67 from Pará state), benzaldehyde, and phenylacetaldehyde also discriminate these beans. Other compounds, although not discriminating, appear important in determining their aromatic quality. The PCA showed that cocoa from Pará state formed a cluster due to similar aromas, while FL89 was the most distinct among the genotypes. Beans from Brazil show great potential and diversity for the fine cocoa market.

## 1. Introduction

The aroma is an essential quality attribute for the commercial valuation of cocoa beans. In addition to the bulk type, a new category known as special cocoa has been established in the international market, including fine cocoa and those characterized by differentiating factors such as origin (*terroir*), certifications, and other singularities [1].

The fine cocoa market has shown a great increase compared to other cocoa segments. This demand has been driven by consumption trends for healthier chocolates, with unique origins and different organoleptic properties (floral, fruity, caramel, and nutty notes) [1].

Currently, Brazil is the sixth-largest producer of cocoa beans in the world, and the states of Pará and Bahia are responsible for this production [2,3]. In 2020, the International Cocoa Organization (ICCO) officially recognized Brazil as a producer and exporter of fine cocoa, expanding the opportunities to increase its competitiveness in the international market.

The origin and genotype of cocoa directly influence the amount and proportion of essential molecules for the optimal production of aromatic compounds [4]. Traditionally, there are three varieties of cocoa: Criollo, Forastero, and Trinitario. The latter is a result of a cross between Criollo and Forastero beans. However, Motamayor et al. [5] using 96 microsatellite markers, observed that there is a significant genetic variation within the Forastero group, and a new subclassification was suggested: Marañon (e.g: PA), Curaray (AGU), Criollo, Iquitos (IMC), Nanay (NA), Contamana (SCA), Amelonado (BE), Purús (CAB), Nacional (MO), and Guyana (CJ) [5].

This new division more accurately reflects the genetic diversity of the species. However, this classification is based only on genetic similarities, not morphological characteristics. Various sizes, colors, shapes of pods, and beans can occur within the same genetic group. Forastero beans usually have a purple color due to the presence of anthocyanins (beans with a stronger and more astringent flavor). At the same time, the white or ivory colors usually attributed to the rare Criollo cocoa (mild nutty flavors) and some Trinitario beans (e.g., ICS95) can also be found in the Forastero Catongo and Almeida beans (absence of the gene coding for the synthesis of anthocyanins). The Nacional genotype has light purple seeds and produces the *arriba* flavor with floral and spicy aromatic notes.

Terpenoids, such as linalool, geraniol, and geranyl acetate are naturally genotype dependent. For example, linalool concentrations reach between 90 and 146 µg/kg in Nacional cocoa liquor [6]. In Brazil, specific peculiar sensory profiles emerged from local experiments involving specific flavor descriptors of cocoa beans, such as whisky, milk chocolate, tangerine, or citrus notes.

In addition to genetics, the aroma is considerably influenced by the post-harvest processing steps (fermentation, drying, roasting, and conching), which cause biochemical reactions within the seed and allow for the formation of flavors, color precursors, the reduction of bitterness, and astringency, improving the sensory characteristics [4].

The formation of flavor precursor compounds occurs during fermentation, as well as a significant increase in volatile compounds such as organic acids (lactic and acetic acids), alcohols, esters, aldehydes, and ketones, well-known for their aromatic properties [1]. Due to the great importance of fermentation, fast and non-destructive methods have been developed to evaluate the cocoa bean fermentation levels [7]. Roasting and conching will also influence the final perception of chocolate through the production of pyrazines, furans, and Strecker aldehydes [8,9].

The aim of this work was to extend our chemical knowledge of fermented cocoa bean flavors, investigating Brazilian samples with very specific and unusual sensory descriptors from the states of Bahia and Pará. Solvent-assisted flavor evaporation (SAFE) was applied to recover representative aroma extracts for GC-EIMS analysis.

## 2. Results Discussion

An analysis of the volatile fraction of unroasted Catongo and FL89 cocoa beans allowed for the classification of the characteristic aromas of each genotype. A few aromas were found at significantly higher levels in Catongo, while others were found in FL89. In addition to compounds appearing as specific to one genotype or the other, some emerged as non-discriminating. The molecules discussed in this section are suspected “odor-active” ones whose concentrations exceed their respective perception thresholds. The results obtained were subjected to a statistical analysis to highlight compounds whose concentrations are significantly different between different cocoas. The Student-Newman-Keuls test was carried out at two levels: it first took into account only the cocoas from Bahia (Catongo and FL89), and then, in a second step, it also included the results obtained for the three cocoas from the Pará region (IMC67, P7, and PA121). Normalized values (a range given by one, two, or three asterisks next to OAVs in the following tables) allow us to determine, among the five genotypes, specific compounds with the most significant impact.

### 2.1. Characteristic Aromas of Catongo

As depicted in Table 1, only three esters among the molecules identified as odor-active were found at significantly higher concentrations in Catongo than in FL89. However, they are representative of the expected aroma of this cocoa, whose known specificity is a kerosene-like aroma tending towards a solvent sensation, despite its red fruit and dried fruit aromas, which are quite pleasant in the mouth.

Two of these esters, ethyl acetate and isobutyl acetate (OAV: 6.4 and 19, respectively), develop ethereal aromas [13] and may thus be linked to the solvent aspect of Catongo. Although the effect should be less pronounced, both should still be odorant in FL89 (OAV: 2.8 and 9.5, respectively).

When the three cocoas from Pará were included in the Student-Newman-Keuls test, the difference in ethyl acetate concentration between Catongo and FL89 no longer appeared significant. Ethyl acetate for P7 and P121 showed values lower than Catongo and higher than FL89. Isobutyl acetate emerged as a discriminant compound for the two Bahian cocoas, with all three cocoas from Pará displaying significantly lower levels of this ester. Notably, this compound is known to arise from alcoholic fermentation [14], and its presence above its perception threshold in both Bahian cocoas may be linked to the post-harvest process applied on the farm. The analysis of normalized values shows that this compound had a special contribution to Catongo, presenting OAV 2.3 times above the standard deviation for the average of the five genotypes.

In a study where ethyl acetate was quantified in unroasted Trinitario beans, its level was found to depend on the fermentation time [15], peaking at 1120–2210 µg/kg after 24 h of fermentation. The same experiment was carried out with isobutyl acetate, which reached only 190 and 160 µg/kg under the same conditions and 260 µg/kg in non-fermented beans [15].

The third compound found in a higher amount in Catongo was ethyl butanoate, with an OAV of 16 (versus 6 for FL89). Present far above its threshold, it may be involved in the fruity side of this white genotype. P7 and PA121 likewise displayed relatively high concentrations of ethyl butanoate (20–22 µg/kg in P7 and PA121, not significantly higher than in Catongo).

### 2.2. Characteristic Aromas of FL89

The aroma of FL89 is associated with that of whisky. Table 2 shows the levels of aroma compounds found at a higher concentration in FL89 than in Catongo. They include four esters, two alcohols, three 7- or 9-C carbonyls, one terpenoid, two aromatic alde-hydes, and three Strecker aldehydes.

Esters such as isoamyl propionate, ethyl heptanoate, ethyl octanoate, and ethyl benzoate should bring fruity/alcoholic beverages aromas to the FL89 cocoa. These esters also appeared odor-active in Catongo, although present at lower concentrations than in FL89. The statistical comparison with IMC67, P7, and PA121 showed their levels to also be significantly lower in the cocoas from Pará. This confirms them as relatively specific to FL89 among the five Brazilian genotypes considered.

Notably, ethyl benzoate has been reported as a contributor to the nutty character of malt whisky [20]. Despite its relatively low OAV in FL89, this compound remains odor-active and may thus be involved in the specific whisky-like aroma of this Bahian genotype.

Ethyl octanoate appears as the most odor-active ester characteristic of FL89. It should develop pineapple, waxy, and alcoholic-beverage-like flavors [13]. An analysis of Criollo beans [21] has highlighted a range of 33 to 165 µg/kg for this compound in Haitian, Malagasy, and Mexican unroasted Criollo. The cocoas from Pará are within this range, but the concentration found in Comoros Criollo beans (described as cognac-like) still exceeds the level found in Brazilian FL89 beans: 271 and 102 µg/kg, respectively. The confirmation of an involvement of ethyl octanoate in some fine cocoas has been obtained with cocoa liquor made from Nacional beans (concentrations up to 140 µg/kg) [6].

Ethyl heptanoate also appeared more abundant in FL89 (statistically equal (*p* < 0.05) to PA121), and this may partly explain the whisky-like aspect of this cocoa, since ethyl heptanoate is known to develop a cognac/rummy/winey aroma [13]. Its concentration in FL89 is also higher than that previously found in Haitian, Mexican, and Malagasy unroasted Criollo beans [21]. Higher concentrations were found only in the genetically verified Haitian Criollo SHA068 (14 µg/kg) and in Criollo from the Comoros (33 µg/kg).

Heptan-2-ol at 1160 µg/kg has been highlighted in unroasted Criollo beans from Grenada [17], its concentration being only slightly lower than that found in FL89. A much higher concentration still (2400 µg/kg) has been reported for a Nacional cocoa liquor [6]. Heptan-2-ol (1330 µg/kg) and heptan-2-one (834 µg/kg), with respective OAVs of 4.4 and 83.4 in FL89, should bring fruity/blue cheese aromas to the FL89 cocoa.

Most cocoa alcohols are known to be produced through fermentation. Yet 1-octen-3-ol, which emerged here as a discriminant for FL89, may be genotype dependent. With 37 µg/kg (OAV = 37), this compound, known to develop a mushroom aroma, has been reported as a contributor to the green odor of Scotch malt whisky, like *trans*-2-nonenal [20]. Its concentration was found to exceed both those recorded for cocoa from Pará and the low range (3 µg/kg to 6 µg/kg) quantified in unroasted Criollo beans [21]. The amount present in unroasted beans is liable to be significantly modified through chocolate processing, as in the case of other alcohols that can be chemically degraded or volatilized during roasting [22].

Most of odor-active seven- and nine-carbon chain compounds identified also tended to be more abundant in FL89.

Heptan-2-one appeared equally concentrated in FL89 and P7 at a level exceeding those found in the other three Brazilian cocoas. Its concentration in FL89 (834 µg/kg) is higher than that previously reported for Nacional cocoa liquor (up to 560 µg/kg) [6].

*Trans*-2-Nonenal was identified as another highly active compound in FL89. Known to impart the unpleasant cardboard aroma to aged beers but also imparting a typical green odor to Scotch malt whisky [20], it could most probably be involved in the whisky-like aroma of the FL89.

The main difference observed between the two Bahian cocoas concerns cheesy-like nonan-2-one (904 µg/kg in FL89 vs. 3.3 µg/kg in Catongo). The concentration found in FL89 is also higher than the 250 µg/kg reported in Nacional cocoa liquor [6]. The observed concentrations of both *trans*-2-nonenal and nonan-2-one exceed the range reported for roasted and unroasted Criollo beans [17,21].

Terpenoid quantification revealed only one discriminant compound in FL89: linalool, at 238 µg/kg (vs. 14 µg/kg in Catongo). With an OAV of 6.4, this compound could develop flowery notes in FL89. The statistical comparison with cocoa samples from Pará showed even higher concentrations in IMC67 (910 µg/kg). The range found in samples of cocoa from Pará (297–910 µg/kg) remains above the concentrations for unroasted Criollo beans from Haiti (23–61 µg/kg) [21], and Grenada (120 µg/kg) [17]. However, processed cocoa showed lower linalool concentrations, with 146 µg/kg found in Nacional cocoa liquor and only 20 µg/kg found in Nacional chocolate [18].

Among the aldehydes with an aromatic ring, benzaldehyde and phenylacetaldehyde proved to be odor-active with a significantly higher concentration in FL89 (3163 µg/kg, OAV = 53 for benzaldehyde and 1042 µg/kg, OAV = 47 for phenylacetaldehyde). Both were found at significantly lower concentrations in cocoas from Pará.

The high level of benzaldehyde (almond/fruity aroma) found in FL89 is not common in cocoa (<2200 µg/kg in unroasted Trinitario from Ecuador, <1613 µg/kg in unroasted Criollo beans from Madagascar, <573 µg/kg in unroasted Criollo beans from Mexico, <2151 µg/kg in unroasted Criollo beans from Comoros, and <1016 µg/kg in most unroasted Haitian Criollo beans) [15,21]. The only previously reported exception is one sample of Criollo beans from Haiti, with 4921 µg/kg [21]. The concentration found in Nacional cocoa liquor, reaching up to 830 µg/kg, is closer to the 821 µg/kg found in Catongo [6]. 

As for phenylacetaldehyde (flowery), its level in FL89 exceeds those found in unroasted Criollo (440–930 µg/kg) [21] and unroasted Trinitario beans [15].

Surprisingly, already before roasting, some Strecker aldehydes appeared abundant in FL89: 2-methylbutanal (3900 µg/kg), 3-methylbutanal (2196 µg/kg), and methional (289 µg/kg). These are usually formed during roasting by Strecker degradation of free amino acids [22], along with pyrazines, which did not reach their perception thresholds in the cocoa beans investigated here. It is most likely that the detected Strecker aldehydes were synthesized during the drying step just after fermentation (7 days in the sun) and, to a lesser extent, during storage and fermentation of the beans. A previous report mentions levels not exceeding 230 µg/kg in unroasted Trinitario from Ecuador [15], values closer to those found for cocoas from Pará.

As confirmed by the normalized OAV values often above 2, most of the here-discussed volatiles have a higher impact on the FL89 genotype, except for linalool, which shows the highest value in IMC67.

### 2.3. Non-Discriminating Aromas

As depicted in Table 3, besides the compounds reported as characteristic of Catongo or FL89, some molecules whose concentrations do not differ significantly between these two genotypes are worth discussing because of their OAVs well above one unit.

Among these compounds, 3-methylbutyl acetate and 2-methylbutyl acetate (banana-like) are common to all the cocoa genotypes analyzed (no statistically significant difference between the five genotypes).

The measured concentration of honey-like 2-phenylethyl acetate was higher in both Bahian cocoas than in those from Pará.

Methyl anthranilate was found predominantly in Bahian cocoas and in IMC67, and P7. PA121 was the only cocoa where the concentration of this compound was significantly lower than in the two Bahian cocoas. Myrcene emerged as another compound exceeding its threshold in both cocoas from Bahia (OAV = 1−3.2) without showing any significant concentration difference between them. Likewise, the test including the Pará genotypes showed no significant difference in myrcene concentration between any two genotypes.

3-Methylbutanol and 2-phenylethanol both exceeded their perception thresholds in the investigated Bahian cocoas. These two compounds originate from fermentation. Fruity-/whisky-like 3-methylbutanol, although not significantly more concentrated in FL89, could be involved (OAV = 17) in the whisky-side aroma of this cocoa. The comparison with the three Pará cocoas did not show any significant difference between the Pará and Bahian cocoas.

Although not discriminant between Bahian cocoas analyzed alone, 2-phenylethanol was found to be significantly more concentrated (3057 µg/kg) in FL89 than in the three cocoas from Pará while also exceeding the concentration range reported for unroasted Criollo beans from Haiti, Madagascar, and Mexico, as well as unroasted Trinitario beans [15,21]. Therefore, 2-phenylethanol could be involved in a more pronounced floral aroma of FL89. Higher values have been found only in Criollo beans from Comoros (6079 µg/kg) [21] and Grenada (3500 µg/kg) [17], as well as in cocoa liquor derived from Nacional beans (3790 µg/kg) [6].

Dimethyltrisulfide and acetoin are two other odor-active compounds, most probably issued from fermentation, whose levels could neither distinguish FL89 from Catongo nor the Bahian cocoas from the Pará cocoas. Acetoin emerged as the most concentrated active compound in all samples, reaching 10,447 µg/kg in FL89 and 17,948 µg/kg in Catongo (the corresponding OAVs were only 13 and 22 because of its high threshold). Lastly, the floral-like acetophenone, which may be a plant metabolite, was found at similar concentrations in the two Bahian genotypes, although the difference between them was found to be significant when the statistical test, including the cocoas from Pará, was applied.

### 2.4. Principal Component Analysis (PCA)

**Figure 1:**
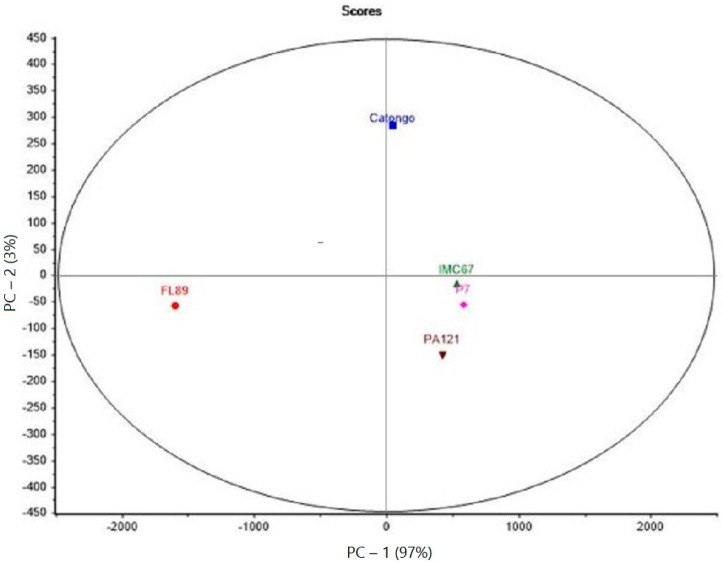
PCA of the five Brazilian cocoa genotypes.

The data set used for the PCA analysis was based on the OAV values of all aroma compounds for the five genotypes (Figure 1).

Two principal components allowed the description of 100% of the total variance of the five studied genotypes. For FL89, it is clearly separated from the other genotypes by PC-1.

All three cocoa samples from Pará presented a cluster (on the right of PC-1), indicating little differentiation (PC-2) among the here-investigated aromas between them.

As for PC1, the Catongo sample revealed to be closer to the Pará genotypes than to FL89, although it comes from the same Bahian producer. This great distinction between FL89 and the others was also evidenced by all the asterisks in Table 2.

## 3. Materials and Methods

### 3.1. Chemicals

Dichloromethane, ethyl acetate, absolute ethanol (99%), and anhydrous sodium sulfate were purchased from VWR International (Leuven, Belgium). Standards of ethyl benzoate, methyl anthranilate, phenylacetaldehyde, 2-methylbutanal, 3-methylbutanal, heptan-2-one, heptan-2-ol, 2-methylbutyl acetate, trans-2-nonenal, methional, isobutyl acetate, benzaldehyde, myrcene, linalool, ethyl heptanoate, ethyl octanoate, 2-phenylethyl acetate, ethyl acetate, ethyl butanoate, isoamyl propionate, acetophenone, and 2-acetylthiophene were purchased from Sigma-Aldrich (Bornem, Belgium). Standard of nonan-2-one was purchased from Janssen Chimica (Geel, Belgium). Standards of dimethyltrisulfide and 1-octen-3-ol were purchased from Acros Organics (Geel, Belgium). Standard of 3-methylbutan-1-ol was purchased from Merck Millipore (Burlington, NJ, USA). Standard of acetoin was purchased from Supelco (Bellefonte, PA, USA).

### 3.2. Cocoa Samples

The Catongo and FL89 cocoas from Bahia were selected because they develop very distinct and promising aromas of whisky (FL89) and oil (Catongo). The Forastero cocoas from Pará (IMC67, PA121 and P7) were selected for their economic importance and productivity in Amazon region.

Fermented, unroasted Catongo and FL89 cocoa beans were provided by the Leolinda farm from Ilhéus (Bahia state, Brazil), where they were individually fermented for 7 days in a wooden box. The other Brazilian cocoa beans (IMC67, PA121, and P7) were obtained from the experimental center of Executive Committee of Cocoa Plantation Plan (Comissão Executiva do Plano da Lavoura Cacaueira–CEPLAC) in Medicilândia (Pará State, Brazil), where they were fermented for 7 days in a wooden box, each genotype being placed in a nylon net to prevent mixing between them. All five genotypes were sun-dried for 7 days until reaching 8% moisture.

### 3.3. Flavor Extraction by Solvent Assisted Flavor Evaporation (SAFE)

The SAFE method described by Engel et al. [24] slightly adapted for cocoa bean powder, was applied to unroasted cocoa beans to extract and quantify all flavors of interest.

The SAFE extraction conditions were as follows: the water bath temperature was set at 40°C, the pressure was kept below 1 × 10^−4^ mbar, and the temperature of the apparatus body (Glasblaeserei Bahr, Manching, Germany) was 30 °C. Two hundred and fifty microliters of a solution containing 2-acetylthiophene at 10 mg/L (IST) were added to ground cocoa beans (50 g) mixed with 50 mL Milli-Q water to obtain an IST concentration of 50 µg/kg. Since the distillation involves cocoa powder, which cannot be introduced dropwise like beer samples, for instance [25], the entire solution was directly introduced into the vessel before applying a vacuum. After 45 min of distillation, the distillate was recovered in a liquid-nitrogen-cooled flask and then extracted three times with 16 mL of biodistilled dichloromethane. The combined organic phases were dried over anhydrous sodium sulfate, concentrated to 0.5 mL in a Kuderna-Danish apparatus at 45 °C and stored at −80 °C prior to gas chromatography-electron impact mass spectrometry (GC-EIMS) analysis.

### 3.4. GC-EIMS for the Quantitation of Flavors of Interest

One microliter of each SAFE extract was analyzed with an Agilent 7890 B gas chromatograph equipped with a splitless injector maintained at 250 °C. The split vent was opened after 0.5 min. Compounds were separated with a WCOT capillary column (CP-Sil 5 CB, 50 × 0.32 mm i.d., 1.2 μm film thickness). The carrier gas was helium, and the pressure was set at 55 kPa. The oven temperature was programmed to rise from 36 °C to 85 °C at 20 °C/min; then, to 145 °C at 1 °C/min; then, to 220 °C at 3 °C/min; and finally, to 250 °C at 30 °C/min. Then it is held at this temperature for 30 min. The column was connected to a single quadrupole mass spectrometer (Agilent 5977 B MSD) operating in full-scan or single-ion monitoring (SIM) mode with electron ionization at 70 eV. Chromatograms were recorded throughout elution. Agilent OpenLab software was used to process the resulting data. Calibration curves (with areas relative to IST) were constructed for compounds of interest, and the following equation was used for the quantitation of compound A: concentration of A in cocoa (in µg/L) = IST concentration in cocoa (in µg/L) × (A area/IST area) × (IST response coefficient/A response coefficient) × (IST recovery factor/A recovery factor). The IST-relative recovery factor was set at 1 for all compounds (experimental values from 0.7 to 1.1, determined beforehand by standard addition).

Concentrations of volatile compounds were compared with odor threshold values (OTVs) from the literature [10,11,12,16,17,18,19,23].

### 3.5. Statistical Analysis

Each analysis was performed in duplicate. The results available for each cocoa were statistically processed with the SAS Studio program. A Student-Newman-Keuls test (at a significance level of 95%) was applied to discern flavors with significantly different concentrations between the analyzed samples. An exploratory principal component analysis (PCA) was applied on the aroma data of the five genotypes using the Unscrambler X 10.4 software and the NIPALS algorithm. The normalization of the OAV data was performed to compare the impact of each compound between genotypes. One, two, and three asterisks (*,**,***) were assigned when the impact was 1.2 to 2.0 times, 2.1 to 2.9 times, and above 3.0 times higher than the normalized value (1.0), respectively.

## 4. Conclusions

An analysis of the aroma compounds of the two cocoas originating from the Bahia region (Brazil) has revealed compounds that may be linked to the specific olfactory descriptors of these cocoas. Other compounds, although not discriminating, appear important in determining the aromatic qualities of these two cocoa bean genotypes.

Catongo generally showed lower aroma concentrations than FL89. Only three esters were found to be more abundant. Its surprising solvent/kerosene aroma may be due to relatively high concentrations of isobutyl acetate and ethyl acetate, while ethyl butanoate might accentuate its fruity aroma.

The characteristic whisky aroma of FL89 may be partly due to certain compounds associated in the literature with whisky. Examples are *trans*-2-nonenal and 1-octen-3-ol (which show strong aromatic activity in this cocoa), esters such as ethyl benzoate, ethyl heptanoate, and octanoate, and 3-methylbutanol. Heptan-2-ol, heptan-2-one, nonan-2-one, benzaldehyde, and phenylacetaldehyde also discriminate these beans. Although already found to be the most involved in the aromas of unroasted FL89 cocoa, Strecker aldehydes (2-methylbutanal, 3-methylbutanal, and methional) should arise through cocoa roasting during the chocolate-making process.

Alongside the characteristic aromas of each cocoa, some compounds appear important for both genotypes without being specific to one or the other. Among the molecules likely to be most involved in the aromas of both cocoa genotypes, our results highlight 3- and 2-methylbutyl acetate, both responsible for a fruity side. As acetoin emerged as the most concentrated odor-active compound in both genotypes, it should have an impact despite its high threshold.

The PCA showed that FL89 was the most distinct among the five genotypes. Cocoas from Pará appeared as a cluster in this analysis. Despite being classified as Forastero, they showed many key-flavors at levels comparable to those found in FL89, with even higher concentrations of linalool for IMC67.

Subsequently, we have the project of analyzing the 20 genotypes of cocoa beans produced in Pará (the largest cocoa producer in Brazil) and evaluating their potential for fine aromas, in addition to distinguishing these genotypes based on other parameters such as phenolic compounds, alkaloids, and physico-chemical composition.

## Figures and Tables

**Table 1:** GC-EIMS quantification of volatile aroma compounds characteristic of Catongo. Concentrations given in µg/kg and followed by Odor Activity Values (OAV) in parentheses.

Chemical Group	Molecule	RI (CPSil-5)	Odor Description	OTV ^+^	Cocoas from Bahia		Cocoas from Pará		
					Catongo	FL89	IMC67	P7	PA121
Esters	Ethyl acetate	600	Fruity/ethereal	940 [10]	6014 ^a,i^ (6.4)	2613 ^b,i,j^ (2.8)	773 ^j^ (0.8)	4153 ^i,j^ (4.4)	5809 ^i^ (6.2)
	Ethyl butanoate	741	Fruity	1 [11]	16 ^a,i^ (16)	6 ^b,j^ (6)	nd	20 ^i^ (20)	22 ^i^ (22)
	Isobutyl acetate	792	Fruity/ethereal	66 [12]	1225 ^a,i^ (19) **	628 ^b,j^ (9.5)	7 ^k^ (0.1)	23 ^k^ (0.3)	18 ^k^ (0.3)

^+^ OTV, odor threshold value (µg/kg). Different superscripts in a row (^a^ or ^b^) point out significant differences (*p* < 0.05) between Catongo and FL89. Superscripts ^i,j,k^ have the same meaning, but for the statistical analysis, including cocoas from Pará. ** Normalized value of OAV significantly (2.1 to 2.9 times) higher.

**Table 2:** GC-EIMS quantification of volatile aroma compounds characteristic of FL89. Concentrations given in µg/kg and followed by Odor Activity Values (OAV) in parentheses.

Chemical Group	Molecule	RI (CPSil-5)	Odor Description	OTV ^+^	Cocoas from Bahia		Cocoas from Pará		
					FL89	Catongo	IMC67	P7	PA121
Esters	Isoamyl propionate	952	Banana, pineapple	9 [12]	18 ^a,i^ (2) *	7.1 ^b,j^ (0.8)	3.1 ^j^ (0.3)	2.7 ^j^ (0.3)	2.9 ^j^ (0.3)
	Ethyl heptanoate	1079	Fruity, cognac	2 [16]	6.2 ^a,i^ (3.1)	2.3 ^b,j^ (1.1)	1.2 ^k^ (0.6)	3.5 ^j^ (1.7)	5.7 ^i^ (2.8)
	Ethyl benzoate	1150	Dry fruits	60 [11]	99 ^a,i^ (1.7)	73 ^b,j^ (1.2)	32 ^l^ (0.5)	32 ^l^ (0.5)	42 ^k^ (0.7)
	Ethyl octanoate	1180	Fruity, floral, alcoholic beverage	5 [16]	102 ^a,i^ (20) *	33 ^b,k^ (6.6)	38 ^k^ (7.6)	64 ^j^ (13)	70 ^j^ (14)
Alcohols	Heptan-2-ol	887	Citrusy	300 [17]	1330 ^a,i^ (4.4) *	123 ^b,k^ (0.4)	219 ^k^ (0.7)	699 ^j^ (2.3)	263 ^k^ (0.9)
	1-Octen-3-ol	962	Mushroom	1 [10]	37 ^a,i^ (37) *	13 ^b,j^ (13)	14 ^j^ (14)	8.3 ^k^ (8.3)	7.9 ^k^ (7.9)
7 or 9-C carbonyles	Heptan-2-one	869	Fruity, floral, blue cheese	10 [18]	834 ^a,i^ (83.4)	298 ^b,j^ (30)	445 ^j^ (44)	682 ^i^ (68)	391 ^j^ (39)
	Nonan-2-one	1072	Cheesy	100 [18]	904 ^a,i^ (9) ***	3.3 ^b,j^ (0.03)	25 ^j^ (0.2)	123 ^j^ (1.2)	17 ^j^ (0.2)
	*trans*-2-Nonenal	1139	Cardboard	0.1 [16]	5.4 ^a,i^ (54) **	2.0 ^b,j^ (20)	nd	nd	nd
Terpenoid	Linalool	1087	Floral, green	37 [17]	238 ^a,j^ (6.4)	14 ^b,k^ (0.4)	910 ^i^ (24) *	297 ^j^ (8.0)	411 ^j^ (11)
Aldehydes with an aromatic ring	Benzaldehyde	939	Almond	60 [18]	3163 ^a,i^ (53) *	821 ^b,j^ (14)	1238 ^j^ (21)	890 ^j^ (15)	1081 ^j^ (18)
	Phenylacetaldehyde	1018	Honey, floral	22 [17]	1042 ^a,i^ (47) *	376 ^b,j,k^ (17)	452 ^j^ (20)	259 ^j,k^ (12)	151 ^k^ (6.9)
Strecker aldehydes	3-Methylbutanal	633	Chocolate	5.4 [11]	2196 ^a,i^ (407) **	42 3 ^b,j^ (78)	107 ^k^ (20)	97 ^k^ (18)	174 ^k^ (32)
	2-Methylbutanal	643	Chocolate	2.2 [18]	3900 ^a,i^ (1773) **	773 ^b,j^ (351)	284 ^j^ (129)	250 ^j^ (114)	633 ^j^ (288)
	Methional	872	Potato	0.2 [19]	289 ^a,i^ (1446) *	129 ^b,j^ (644)	29 ^k^ (145)	16 ^k^ (80)	23 ^k^ (115)

^+^ OTV, odor threshold value (µg/kg). Different superscripts in a row (^a^ or ^b^) point out significant differences (*p* < 0.05) between Catongo and FL89. Superscripts ^i,j,k^ have the same meaning, but for the statistical analysis, including the cocoas from Pará. *, **, *** Normalized value of OAV significantly higher (1.2 to 2.0 times, 2.1 to 2.9 times, and above 3.0 times, respectively).

**Table 3:** GC-EIMS quantification of non-discriminating volatile aroma compounds. Concentrations given in µg/kg and followed by Odor Activity Values in parentheses.

Chemical Group	Molecule	RI (CPSil-5)	Odor Description	OTV ^+^	Cocoas from Bahia		Cocoas from Pará		
					FL89	Catongo	IMC67	P7	PA121
Esters	3-Methylbutyl acetate	853	Fruity, banana	10 [18]	5085 ^a,i^ (530)	2941 ^a,i^ (306)	4546 ^i^ (455)	4545 ^i^ (455)	5135 ^i^ (513)
	2-Methylbutyl acetate	868	Fruity, banana	5 [12]	671 ^a,i^ (134)	688 ^a,i^ (138)	460 ^i^ (92)	682 ^i^ (136)	746 ^i^ (149)
	2-Phenylethyl acetate	1228	Honey, floral	137 [18]	729 ^a,i^ (5.3)	722 ^a,i^ (5.6)	355 ^k^ (2.6)	545 ^j^ (4.0)	534 ^j^ (3.9)
	Methyl anthranilate	1322	Fruity, orange flower	3 [11]	5.1 ^a,i^ (1.7)	4.7 ^a,i^ (1.6)	3.4 ^i,j^ (1.1)	3.1 ^i,j^ (1.0)	0.5 ^j^ (0.2)
Terpenes	Myrcene	982	Spicy	9.2 [18]	26 ^a,i^ (3.2)	8.9 ^a,i^ (1)	20 ^i^ (2.2)	11 ^i^ (1.2)	3.2 ^i^ (0.3)
Alcohols	3-Methylbutanol	728	Whisky, fruity	100 [10]	1726 ^a,i^ (17)	1484 ^a,i^ (15)	851 ^i^ (8.5)	664 ^i^ (6.6)	891 ^i^ (8.9)
	2-Phenylethanol ^IST^	1088	Floral	211 [17]	3057 ^a,i^ (14)	1995 ^a,j^ (9.4)	1177 ^k^ (5.6)	1000 ^k^ (4.7)	1077 ^k^ (5.1)
Sulfur compounds	Dimethyltrisulfide	959	Cooked onion	0.3 [18]	2.0 ^a,i^ (6.7)	1.0 ^a,i^ (3.3)	11 ^i^ (37)	7.3 ^i^ (24)	12 ^i^ (40)
Ketones	Acetoin	682	Buttery, creamy	800 [23]	10,774 ^a,i^ (13)	17,948 ^a,i^ (22)	11,234 ^i^ (14)	10,768 ^i^ (13)	12,629 ^i^ (16)
	Acetophenone ^IST^	1042	Floral	65 [23]	262 ^a,i^ (4)	232 ^a,j^ (3.6)	169 ^k^ (2.6)	107 ^l^ (1.6)	115 ^l^ (1.8)

^+^ OTV, odor threshold value (µg/kg). Different superscripts in a row (^a^ or ^b^) point out significant differences (*p* < 0.05) between Catongo and FL89. Superscripts ^i,j,k^ have the same meaning but for the statistical analysis, including the cocoas from Pará. Compounds assigned a superscript “^IST^” with are quantified in 2-acetylthiophene equivalents.

## Data Availability

All relevant data are included in the article.

## Sample Availability

Samples of the compounds are available from the authors.
